# Frailty and social participation in older citizens in Japan during the COVID‐19 pandemic

**DOI:** 10.1002/jgf2.539

**Published:** 2022-03-30

**Authors:** Sachiko Ozone, Ryhei Goto, Shogo Kawada, Shoji Yokoya

**Affiliations:** ^1^ Faculty of Medicine University of Tsukuba Tsukuba Japan

**Keywords:** COVID‐19, frailty, Kihon checklist, older, social participation

## Abstract

**Background:**

This study examined the frailty status of older individuals in Japan at 1 year after the onset of the coronavirus disease 2019 (COVID‐19) pandemic based on involvement in social activities before and during the pandemic.

**Methods:**

This cross‐sectional study analyzed citizens aged 65 and 84 who did not require long‐term care in January 2021. A self‐administered questionnaire was mailed to 3000 citizens in Kitaibaraki City, Japan. The questionnaire included social participation status in January 2020 and January 2021, the Kihon Checklist, working status, and economic status. We classified the respondents into the following groups: Nonparticipating, no participation at either time point; Discontinued, participation only in 2020; and Continued, participation at both time points. We compared the Discontinued and Continued groups in terms of Kihon Checklist items using the *t*‐test.

**Results:**

Of 2963 individuals who received the questionnaire, 1307 (44.1%) returned it, and 1047 were analyzed. Of the respondents analyzed, 586 (56.0%) were in the Nonparticipating group, 254 (24.3%) were in the Discontinued group, and 207 (19.8%) were in the Continued group. On the Kihon Checklist, oral function and mood differed significantly between the Discontinued and Continued groups. The proportion of those with impairment in multiple categories of the Kihon Checklist was 12.3% in the Nonparticipating group, 5.5% in the Discontinued group, and 3.4% in the Continued group.

**Conclusions:**

Older individuals who continued participating in social activities at 1 year into the COVID‐19 pandemic might have a lower risk of frailty in terms of oral function and depressed mood.

## Graphical abstract

This study examined the frailty status using the Kihon Checklist of the older citizens in Japan one year after the onset of the COVID‐19 pandemic according to involvement in social activities before and during the pandemic. Of the 1047 participants analyzed, participants who continued with their social participation activities had the lowest proportion of having impairment in multiple categories of the Kihon Checklist.

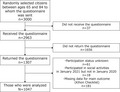

## 1 INTRODUCTION

Social participation is important for maintaining the physical and mental health of older adults. Social participation is reported to be related to hypertension,^1^ cognitive function,^2^ depression,^3^ self‐reported health,^4^ and frailty.^5^, ^6^, ^7^ The World Health Organization (WHO) considers social participation to be a key area for action on healthy aging.^8^

Although social participation is gaining much attention, its definition is diverse. Levasseur et al defined that social participation “mainly focused on the person’s involvement in activities providing interactions with others in the society or the community,” and that involvement could be classified into several levels.^9^ Douglas et al defined three concepts of social participation: social connections, informal social participation, and volunteering. Although these concepts are often discussed interchangeably, they are all related to physical and mental health outcomes.^10^

The coronavirus disease 2019 (COVID‐19) pandemic that began in 2020 led to the limitation of numerous activities worldwide.^11^, ^12^ Studies have reported that decreased involvement in social and physical activities negatively impacts health outcomes.^11^, ^13^ In Japan, the government declared its first pandemic‐related state of emergency from April to May 2020, followed by a second declaration limited to specific areas from January to March 2021.^14^ Although neither state of emergency was legally binding, many social activities were suspended nationwide beginning in April 2020, and physical activity significantly decreased between January and April 2020 among older adults in Japan.^15^

It became particularly difficult for older adults to resume their normal activities as the characteristics of COVID‐19 became clear. Older adults have the highest COVID‐19‐related mortality rates, and those with comorbidities are at particularly high risk of severe disease and death.^16^ Social distancing has been strongly encouraged when resuming activities involving multiple people,^17^ and many online events or activities have taken place instead.^18^ However, it is often difficult for older adults to maintain appropriate distancing in their social activities or to replace them with online alternatives. As a result, even in areas where COVID‐19 infection is somewhat under control, it may be difficult for older people to participate in social activities as they did before the pandemic.

The first COVID‐19 case in Japan was reported on January 16, 2020. On February 27, the government declared a state of emergency from April to May. Ibaraki Prefecture, located to the north of the Tokyo metropolitan area, declared a state of emergency on January 11, 2021, in accordance with the federal government’s region‐limited emergency declaration for the Tokyo metropolitan regions.^14^ Kitaibaraki City is located in Ibaraki Prefecture. It is 140 kilometers north of the Tokyo metropolitan area. It has a population of 41,400, and 35.0% of residents were aged 65 or older as of 2020.^19^ All activities in Kitaibaraki City that involved social interaction were temporarily suspended in April 2020. Some activities resumed in July 2020 after infection prevention measures were instituted. The first case of COVID‐19 in the city was reported in October 2020. A total of 12 cases had been reported as of January 2021.^19^

Suspending social participation for a long time during the COVID‐19 pandemic might greatly increase the frailty of older adults, possibly in ways different from prepandemic times because of difficulties in directly interacting with others as a result of pandemic control measures. Shinohara et al reported that as of May 2020, a COVID‐19‐related decrease in daily movement was related to increased frailty among older adults who were helped by local volunteers in a nonmetropolitan area in Japan.^20^ However, investigating the long‐term effects of decreased daily movement or social participation during the COVID‐19 pandemic was beyond the scope of their study. If we can determine how the health of older adults during the COVID‐19 pandemic relates to their degree of social participation, even in regions with a relatively low number of cases, we might be able to better tailor frailty prevention measures in this population.

The purpose of this study was to clarify the frailty status of older residents in a city where COVID‐19 transmission was relatively under control at 1 year into the COVID‐19 pandemic according to the degree to which they engaged in social participation activities before and during the pandemic.

## 2 METHODS

### 2.1 Settings and participants

In January 2021, we conducted a cross‐sectional questionnaire survey in Kitaibaraki City, Ibaraki Prefecture, Japan.

Individuals eligible to participate in this survey were citizens of Kitaibaraki City whose ages were between 65 and 84 and who were not certified as requiring long‐term care under long‐term care insurance. Approximately 9000 citizens met the criteria, and we randomly selected 3000 of them as participants in this survey.

We mailed a self‐administered questionnaire to these individuals and asked them to mail it back. The questionnaire contained a form on which respondents could provide written informed consent.

### 2.2 Measurements

We asked each respondent for their age, gender, residential status (living alone or not), working status (working or not), satisfaction with economic status (satisfied or not), and social participation status in January 2020 and January 2021. We used the Kihon Checklist (KCL) to determine the frailty status of each respondent. The Kihon Checklist (KCL) is a self‐reported comprehensive health checklist that is used as a screening tool to identify community‐dwelling older adults who are at increased risk of becoming frail.^21^, ^22^ The KCL may predict the future need for long‐term care insurance in Japan.^23^ The instrument consists of 25 items divided into seven categories: physical strength, nutrition, eating, socialization, memory, mood, and lifestyle. We analyzed the responses for each category and also, after excluding the five items related to mood, identified respondents who scored positively on 10 or more of the remaining 20 items. These individuals were considered to demonstrate “impairment on multiple items,” which is one of the criteria used by the Japanese Ministry of Health, Labor and Welfare (MHLW) to identify older adults who are at increased risk of becoming dependent.^21^ We also evaluated each category according to the criteria set by MHLW.^21^

We defined social participation based on the regional characteristics of Kitaibaraki City. We asked the respondents “Were you participating in either local festivals or events, community activities in the neighborhood, voluntary groups, or volunteer activities in January 2020?” We also asked about specific activities in which they participated using a multiple‐choice format. We asked the same questions about their social participation status in January 2021. We categorized respondents into three groups according to their participation status: 1) Nonparticipating group, those who did not engage in social participation activities in either January 2020 or January 2021; 2) Discontinued group, those who participated in January 2020 but not in January 2021; and 3) Continued group, those who participated in both January 2020 and January 2021. Since the focus of this study was on nonparticipation, discontinuation, and continuation of social participation and not on initiation, we excluded respondents who did not know whether they engaged in social participation activities, those who did not participate in January 2020 but did participate in January 2021, and those who did not answer all Kihon Checklist items.

### 2.3 Statistical analysis

The main outcome of the study was difference in frailty status between those who continued and discontinued social participation activities during the COVID‐19 pandemic based on the KCL. After providing descriptive data on the three groups, we compared the Discontinued and Continued groups in terms of age, gender, residential status, working status, and KCL categories using the *t*‐test or the chi‐squared test. We analyzed the data using IBM SPSS Statistics for Windows, version 27 (IBM Corp.). *p* values below 0.05 indicated statistical significance. The study was approved by the University of Tsukuba Medical Ethics Board (number 1602).

## 3 RESULTS

**Figure 1:**
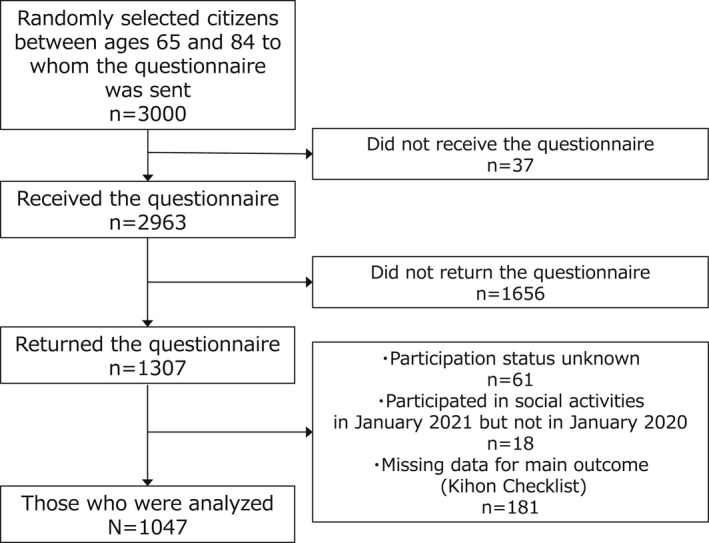
Flowchart of the study participants

Of the 3000 citizens selected, 2963 received the questionnaire and 1307 returned it (response rate, 44.1%). We excluded 61 respondents who did not know whether they engaged in social participation activities, 18 who did not participate in January 2020 but did participate in January 2021, and 181 who did not answer all the items of the Kihon Checklist. As a result, 1047 respondents were analyzed (Figure 1).

The mean age of the respondents was 72.9 (standard deviation, 5.3) years, and 520 (49.7%) were female. In January 2021, 136 respondents (13.0%) lived alone, 316 (30.2%) were working, and 511 (48.8%) were satisfied with their economic status. Of the respondents analyzed, 586 (56.0%) were included in the Nonparticipating group, 254 (24.3%) in the Discontinued group, and 207 (19.8%) in the Continued group (Table 1). The Discontinued and Continued groups had no significant difference in baseline characteristics.

**Table 1:** Respondent characteristics categorized by social participation status^a^ before and during the COVID‐19 pandemic

	Nonparticipating (*n* = 586)	Discontinued (*n* = 254)	Continued (*n* = 207)	*p* value
Age, mean ± SD (years)	73.0 ± 5.5	72.6 ± 5.0	73.3 ± 5.2	0.151^†^
Women, *n* (%)	262 (44.7)	145 (57.1)	113 (54.6)	0.482^‡^
Living alone, *n* (%)	75 (12.8)	30 (11.8)	31 (15.0)	0.322^‡^
Working, *n* (%)	182 (31.1)	74 (29.1)	60 (29.0)	0.291^‡^
Economic status, *n* (%)				
Satisfied	251 (42.8)	133 (52.4)	127 (61.4)	0.245^‡^
Unsatisfied	330 (56.3)	117 (46.1)	79 (38.2)	
Unknown	5 (1.0)	4 (1.6)	1 (0.5)	

Abbreviation: SD, standard deviation.

Social participation status was categorized as follows: Nonparticipating, respondents who did not engage in social participation activities in January 2020 or January 2021; Discontinued, those who engaged in social participation activities in January 2020 but not in January 2021; Continued, those who engaged in social participation activities in both January 2020 and January 2021.

*p* value for comparison of the Discontinued and Continued groups using *t*‐test.

*p* value for comparison of the Discontinued and Continued groups using the chi‐squared test.

The results of the KCL are shown in Table 2. The proportions of individuals with impairment in multiple KCL categories in the Nonparticipating, Discontinued, and Continued groups were 12.3%, 5.5%, and 3.4%, respectively. There was a significant difference between the Discontinued and Continued groups in impaired oral function (*p* = 0.009) and possible depressed mood (*p* < 0.001).

**Table 2:** Kihon checklist responses categorized by social participation status^a^ before and during the COVID‐19 pandemic

	Nonparticipating (*n* = 586)	Discontinued (*n* = 254)	Continued (*n* = 207)	*p* value^†^
Physical strength, *n* (%)	116 (19.8)	34 (13.4)	28 (13.5)	0.965
Nutrition, *n* (%)	68 (11.6)	27 (10.6)	13 (6.3)	0.099
Eating, *n* (%)	199 (34.0)	90 (35.4)	50 (24.2)	0.009
Socialization, *n* (%)	61 (10.4)	11 (4.3)	7 (3.4)	0.061
Memory, *n* (%)	231 (39.4)	86 (33.9)	59 (28.5)	0.218
Mood, *n* (%)	207 (35.3)	95 (37.4)	43 (20.8)	<0.001
Impairment in multiple items, *n* (%)	72 (12.3)	14 (5.5)	7 (3.4)	0.275

Social participation status was categorized as follows: Nonparticipating, respondents who did not engage in social participation activities in January 2020 or January 2021; Discontinued, those who engaged in social participation activities in January 2020 but not in January 2021; Continued, those who engaged in social participation activities in both January 2020 and January 2021.

*p* value for comparison of the Discontinued and Continued groups using the chi‐squared test.

## 4 DISCUSSION

In this study, as much as 24.3% of the respondents discontinued their social participation activities, whereas 19.8% continued activities. We found that older individuals who discontinued their social participation activities during the first year of the COVID‐19 pandemic might have a higher risk of frailty in terms of oral function and mood compared with those who continued their social participation activities during the pandemic. Our study provides new insights into how discontinuation of social participation activities during 1 year of the COVID‐19 pandemic might impact frailty among older individuals, even in a city with relatively good control of the pandemic.

A relatively high proportion of participants discontinued social participation activities at 1 year into the COVID‐19 pandemic. In Japan, approximately 62% of males and 55% of females aged 60 years or older participated in social activities or worked in both 2012 and 2016.^24^ In 2019, the rate of participation in local gatherings or community activities was approximately 45%–48% for those aged 60 years or older.^25^ In our study, approximately 42% participated in social participation activities before the pandemic, which was compatible with previous reports.^24^, ^25^ However, the participation rate decreased markedly to 19.8% after 1 year of the pandemic. It could be said that the COVID‐19 pandemic affected the activities of older residents, which might accelerate the negative impact of not engaging in social participation activities at an unprecedented level.

Oral function and mood were significantly impaired in those who discontinued social participation activities at 1 year into the pandemic, even in a suburban city where the pandemic was relatively under control. Social participation is reported to have a preventive effect on the onset of depression.^26^, ^27^ Among older residents in a district of Tokyo, Okamura et al reported that between 2018 and April 2020, when the first state of emergency was declared, the proportion of people with depressed mood increased from 29% to 38%, and the proportion of people with frailty increased from 10% to 16%.^28^ In our study, the prevalence of depressed mood was 20.8% in the Continued group and 37.4% in the Discontinued group, while it was 24.2% and 35.4% for oral frailty. It was noteworthy that the study participants lived in a city in which the COVID‐19 pandemic was not as serious as in metropolitan areas, with only 29.0 cumulative cases per 100,000 population as of January 2021.^19^ We might be able to say that even in nonmetropolitan cities where the COVID‐19 pandemic was not as serious, discontinuation of social participation activities as a result of the COVID‐19 pandemic might affect the incidence of depressed mood and frailty among older residents. Further investigations to assess longer term effects of COVID‐19 and discontinuation of social participation activities are needed.

There were several limitations to this study. First, the study was conducted in a single city in Japan, and the results may have been affected by factors such as local characteristics of the COVID‐19 epidemic and the extent of resumption of social participation. However, the fact that a certain proportion of older citizens discontinued social participation and were then more likely to become frail, even in a city with relatively few COVID‐19 cases, may be generalizable to other regions. Second, since this was a cross‐sectional survey involving a self‐administered questionnaire, recall bias may have influenced the answers regarding social participation status before the pandemic. However, we asked only simple questions about this status, which should have minimized potential bias.

## 5 CONCLUSIONS

More than half of older residents who participated in social participation activities had discontinued their participation at 1 year into the COVID‐19 pandemic, with higher rates of depressed mood and oral frailty. Further research is needed to clarify longer term effects of the COVID‐19 pandemic and discontinuation of social participation activities.

## CONFLICT OF INTEREST

The authors report no conflicts of interest.
